# Exploring the role of uterine fibroids in promotion of cardiovascular diseases by diabetes exposure: Findings from national health and nutrition examination survey 1999–2006

**DOI:** 10.3389/fcvm.2022.975920

**Published:** 2022-08-09

**Authors:** Bin Li, Zhen Yuan, Yizhi Zhang, Feng Li, Lin Huang, Zhihui Yang, Haiyue Liu, Zuheng Liu

**Affiliations:** ^1^Department of Cardiology, Xiamen Chang Gung Hospital, School of Medicine, Huaqiao University, Xiamen, China; ^2^School of Public Health, Southwest Medical University, Luzhou, China; ^3^Pharmaceutical and Medical Technology College, Putian University, Putian, China; ^4^Xiamen Key Laboratory of Genetic Testing, Department of Laboratory Medicine, The First Affiliated Hospital of Xiamen University, School of Medicine, Xiamen University, Xiamen, China; ^5^The Third Clinical Medical College, Fujian Medical University, Fuzhou, China; ^6^Xiamen Key Laboratory of Cardiac Electrophysiology, Department of Cardiology, Xiamen Institute of Cardiovascular Diseases, The First Affiliated Hospital of Xiamen University, School of Medicine, Xiamen University, Xiamen, China

**Keywords:** cardiovascular diseases, uterine fibroids, diabetes, NHANES, hypertension, heart failure, coronary artery disease

## Abstract

**Objective:**

The relationship between uterine fibroids (UF) and cardiovascular diseases (CVDs) in the diabetes population seemed to remain undetermined in previous studies. This study aims to explore the association between UF and CVDs by using the database from the National Health and Nutrition Examination Survey (NHANES). To further evaluate the connection between UF and CVDs we also tested the potential differences due to diabetes exposure.

**Materials and methods:**

National Health and Nutrition Examination Survey data (1999–2006) were collected and used in this study. A total of 5,509 individuals were included and analyzed. The student’s *t*-test and the chi-squared test were used to explore the demographic characteristic between UF and non-UF groups. Logistic regression analysis was performed to determine the odds ratios of UF and covariates.

**Results:**

Female participants were divided into UF (*n* = 694, 12.60%) and non-UF (*n* = 4,815, 87.40%) groups. The incidence of CVDs in UF patients (*n* = 245, 35.30%) were higher than non-UF individuals (*n* = 776, 16.12%) (*p* < 0.001). In addition, each subtype of CVDs were also different, which contains hypertension (33.29 vs. 15.31%, *p* < 0.001), heart failure (1.59 vs. 0.52%, *p* < 0.01), angina (2.59 vs. 0.62%, *p* < 0.001), heart attack (1.73 vs. 0.58%, *p* < 0.01) and coronary heart disease (1.44 vs. 0.54%, *p* < 0.01). The odds ratios of CVDs according to logistic regression were 2.840 (95% CI: 2.387–3.379) for UF patients (*p* < 0.001), while the odds ratios (ORs) were 1.438 (95% CI: 1.175–1.760) after taking account for the age, body mass index (BMI), diabetes, race, education, and annual family income (*p* < 0.001). In addition, secondary analysis indicated more adverse effects in by UF exposure on CVDs risk among non-diabetes individuals (OR = 1.389, 95% CI = 1.124–1.718, *p* < 0.01) than diabetes patients (*p* = 0.063).

**Conclusion:**

Overall, UFs were positively associated with CVDs, and this effect seems blunted by diabetes exposure.

## Introduction

Cardiovascular diseases (CVDs) is one of the leading cause of death throughout the world. As a significant public health problem, the underlying factors that are linked to the incidence and progress of CVDs need to be identified. Uterine fibroid (UF), also known as uterine leiomyomas, is a tumor derived from the muscle layer of the uterus and affects millions of women (1). It is still controversial whether UFs contribute to the occurrence of CVDs. A previous study from Northern Finland indicated that increased blood lipids or metabolic syndrome are associated with a higher risk of UF (2). Thus, it is plausible that diabetes might be essential in UF, while a population study with 3,789 subjects from America indicated that the presence of type 2 diabetes has a protective effect on UF regardless of the medication intervention (3). Although diabetes is a traditional risk factor for CVDs, the connection between UF and CVDs needs more investigation. In addition, another study on symptomatic UF Dutch women did not find any correlation between UF and cardiovascular risks except for hypertension (4). This discrepancy in conclusions might be due to the difference in regions. In addition, elevated blood pressure is positively associated with UF in a prospective study (5). Thus, UF and hypertension might influence and make cause and affect each other, which possibly suggests a similar clinical risk in these two diseases.

Generally, asymptomatic patients do not need medical treatment, as UF belongs to a benign tumor. However, the etiology of UF is obscured, which contains genetic inheritance, hormones, and abnormal stem cells. Indeed, the hereditary susceptibility of UF is influenced by various hormones (6). Estrogen is one of the recognized factors that promote the growth of fibroids (7), while some studies indicated that growth hormone and prolactin collaborated with estrogen to promote mitosis in UF (8). In addition, these hormones are regulated by the hypothalamic-pituitary axis which probably plays a crucial role in its pathogenesis.

Recent studies have indicated that the renin-angiotensin-aldosterone system (RAAS) participated in the progress of UF and hypertension (9). Angiotensin II induces vasoconstriction and promotes the proliferation of leiomyoma cells (4). In addition, Hana et al. proved that the risks of hypertension were increased in UF patients (4). Although they only reveal the connection between UF and hypertension risks, hypertension is indeed a risk factor for various CVDs. Importantly, some studies demonstrated that Angiotensin-converting enzyme inhibitors reduced the incidence of UF (10). Additionally, Angiotensin II is a robust factor in inducing inflammation response (11), while inflammation seems to be a common factor that influences both CVDs and UF. The inflammatory cells not only invaded the uterus and increased the number of fibrotic cells (12), but also increased vascular constriction.

Here, we investigated the connection between CVDs and UF under the stimulation of diabetes by exploring the data from the National Health and Nutrition Examination Survey (NHANES).

## Materials and methods

### Data source and participants

Data from NHANES 1999 to 2006 was used in the analysis. NHANES provides a national estimation of health information in America by conducting interviews, and medical and laboratory examinations on each participant. UF individuals were verified by asking ‘Told by doctor had UFs’ from the questionnaire of RHQ380. The diagnosis of diabetes was verified from the questionnaire of DIQ010. CVDs patients contained hypertension, heart failure, coronary artery disease, angina, or heart attack. The verification of hypertension is based on the questionnaire from BPQ020, while the verification of heart failure, coronary artery disease, angina, and heart attack was according to the questionnaire of MCQ160B, MCQ160C, MCQ160D, and MCQ160E, respectively.

### Statistical analysis

The data analyses were performed by R version 2.1.1 and SPSS statistical package version 20.0. The quantitative data are exhibited as the mean ± standard deviation (ME ± SD), while the qualitative data are shown as numbers (n) and percentages (%). A chi-squared test was performed to assess the differences between each subtype of CVDs. Logistic regression analysis was used to identify the factors that were independently associated with CVDs. The odds ratios (ORs) were used to calculate the risk factors for CVDs. A *p*-value of < 0.05 was considered statistically significant.

## Results

### Descriptive statistics

**FIGURE 1:**
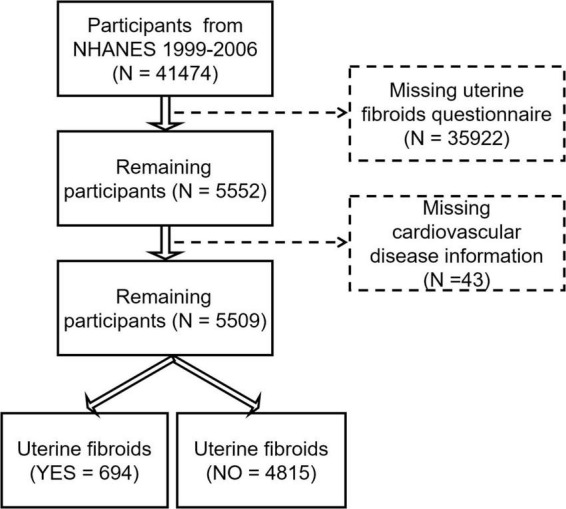
The flowchart of sample selection criteria for the association between cardiovascular diseases (CVDs) and uterine fibroids.

**FIGURE 2:**
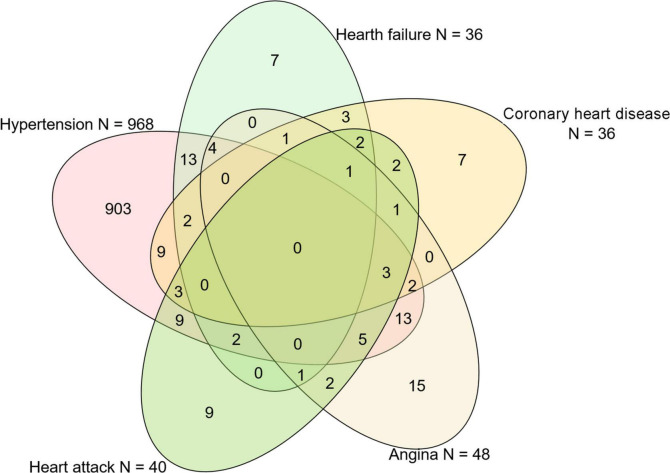
Venn diagram of individuals’ distribution in each subgroup of cardiovascular diseases (CVDs).

In the present study, we enrolled 41,474 participants in NHANES 1999–2006. The definition of CVDs was individuals with hypertension, heart failure, coronary artery disease, angina, or heart attack. After excluding participants without the information of UF (*n* = 35,922) and CVD (*n* = 43), a total of 5,509 females aged from 18 to 54 years old were enrolled (Figure 1). The Venn diagram showed the distribution and overlay of each subtype of CVDs that are enrolled. There are 968 individuals with hypertension, 36 individuals with hypertension, 48 individuals with angina, and 40 individuals with a heart attack in this study (Figure 2). Any individuals without the exact information of the above-described diseases were excluded. Of these included individuals, 2.55% suffered from at least 3 kinds of diseases, 7.65% suffered from at least 2 kinds of diseases, while the percent of hypertension patients suffering from heart failure, coronary artery disease, angina, or heart attack at the same time were 58.33, 55.56, 58.33, and 52.50%, respectively.

### Demographics of participants

Our study included a total of 5,509 participants (Figure 1), in which we identified UF in 694 participants. The clinical characteristics of the participants from NHANES 1999–2006 are reported in Table 1. Approximately 12% (*n* = 694) of the included population are individuals with UF. They were older than the non-UF group, with 70.17% (*n* = 487) of individuals older than 40 years old. In addition, UF individuals have a higher level of body mass index (BMI) (30.50 ± 7.95 vs. 28.62 ± 7.24 kg/m^2^, *p* < 0.001), with almost 45.73% of UF individuals having a BMI larger than 30 kg/m^2^. Compared with non-UF individuals, UF patients that were more likely to suffer from diabetes, had higher education levels, higher family incomes, and higher blood pressure. However, they were no significant differences between smoking status. The morbidity of CVDs in UF patients (35.30%, 245/694) is higher than in non-UF individuals (16.12%, 776/4815). The incidence and subtype of CVDs are shown in Table 1 as numbers and percentages. Then we also explored their difference in each subtype of CVDs, in which, UF individuals have a higher proportion of hypertension, heart failure, angina, heart attack, and coronary heart disease.

**TABLE 1:** Demographics and clinical characteristics of participants [National Health and Nutrition Examination Survey (NHANES) 1999–2006].

Characteristic	Uterine fibroids (*n* = 694, 12.60%)	Non-uterine fibroids (*n* = 4815, 87.40%)	*P-value*
**Age, years, *N*** (%)			
<30	43 (6.20%)	1998 (41.50%)	<0.001^a^
30–40	164 (23.63%)	1361 (28.27%)	
> = 40	487 (70.17%)	1456 (30.24%)	
Mean ± SD	43.65 ± 7.35	34.53 ± 9.90	<0.001^b^
BMI, kg/m^2^, *N* (%)			
<20	30 (4.34%)	331 (6.95%)	<0.001^a^
20–25	166 (24.02%)	1367 (28.69%)	
25–30	178 (25.76%)	1370 (28.75%)	
> = 30	316 (45.73%)	1694 (35.55%)	
Mean ± SD	30.50 ± 7.95	28.62 ± 7.24	<0.001^b^
Missing	1 (0.14%)	3 (0.06%)	
Diabetes, *N* (%)	63 (9.08%)	187 (3.88%)	<0.001^a^
**Race, *N* (%)**			
Mexican American	86 (12.39%)	1205 (25.03%)	<0.001^a^
Other Hispanic	23 (3.31%)	244 (5.07%)	
Non-Hispanic White	297 (42.80%)	2262 (46.98%)	
Non-Hispanic black	264 (38.04%)	879 (18.26%)	
Other race or multi-racial	24 (3.46%)	225 (4.67%)	
Education, *N* (%)			
<High school	117 (16.86%)	1201 (24.97%)	<0.001^a^
High school	149 (21.47%)	1082 (22.49%)	
>High school	428 (61.67%)	2527 (52.54%)	
Missing	0	5 (0.01%)	
**Annual family income, *N* (%)**			
<20,000 USD	140 (20.17%)	1358 (28.20%)	<0.001^a^
≥20,000 USD	532 (76.66%)	3317 (68.89%)	
Missing	22 (3.17%)	140 (2.91%)	
**Current smoking status, *N* (%)**			
Smoking	158 (22.77%)	1048 (21.77%)	0.126^a^
Non-smoking	138 (19.88%)	755 (15.68%)	
Missing	398 (57.35%)	3012 62.55%)	
Cardiovascular disease, *N* (%)	245 (35.30%)	776 (16.12%)	<0.001^a^
Hypertension, *N* (%)	231 (33.29%)	737 (15.31%)	<0.001^a^
Hearth failure, *N* (%)	11 (1.59%)	25 (0.52%)	<0.01^a^
Angina, *N* (%)	18 (2.59%)	30 (0.62%)	<0.001^a^
Heart attack, *N* (%)	12 (1.73%)	28 (0.58%)	<0.01^a^
Coronary heart disease, *N* (%)	10 (1.44%)	26 (0.54%)	<0.01^a^
SBP, Mean ± SD	121.66 ± 18.22	113.56 ± 14.59	<0.001^b^
DBP, Mean ± SD	74.27 ± 11.13	67.85 ± 12.14	<0.001^b^

Data are presented as N% (χ2 test) and mean ± standard deviation (SD) (independent t-test), which are denoted by ^a^ and ^b^, respectively.

### Association between uterine fibroids and cardiovascular diseases

Table 2 shows the crude and adjusted odds ratios for the association between UF and CVDs. Compared to individuals without UF, these UF patients had higher odds ratios for CVDs (OR = 2.840, 95% CI = 2.387–3.379, *p* < 0.001). In addition, this relationship persisted after adjusting for age, BMI, diabetes, race, education, annual family income (OR = 1.438, 95% CI = 1.175–1.760, *p* < 0.001). In the adjusted model, except for education level, age, BMI, race, and family income were significantly associated with CVDs. It is no surprise that diabetes patients have the highest odds ratio for CVDs in the adjudged model (OR = 3.308, 95% CI = 2.459–4.452). Then we explored the relationship between UF and CVDs and presented the results stratified by the history of diabetes.

**TABLE 2:** Logistic regression analysis of uterine fibroids and cardiovascular diseases (CVDs) in National Health and Nutrition Examination Survey (NHANES) 1999–2006.

	OR	95% CI	*P-value*
**Crude model**			
Uterine fibroids			<0.001
Non-uterine fibroids	Ref.	Ref.	
Uterine fibroids	2.840	2.387–3.379	
**Adjudged model**			
Uterine fibroids			<0.001
Non-uterine fibroids	Ref.	Ref.	
Uterine fibroids	1.438	1.175–1.760	
Age, years	1.074	1.065–1.084	<0.001
BMI, kg/m^2^	1.066	1.056–1.077	<0.001
Diabetes			<0.001
Yes	3.308	2.459–4.452	
No	Ref.	Ref.	
Race			<0.001
Mexican American	0.930	0.595–1.453	0.749
Other Hispanic	0.979	0.563–1.702	0.939
Non-Hispanic white	1.272	0.834–1.939	0.264
Non-Hispanic black	1.793	1.162–2.767	<0.01
Other race or multi-racial	Ref.	Ref.	
Education			0.064
<High school	Ref.	Ref.	
High school	1.081	0.858–1.361	0.510
>High school	0.866	0.702–1.070	0.183
Annual family income			<0.01
<20,000 USD	Ref.	Ref.	
≥20,000 USD	0.785	0.656–0.938	

### Association analysis of unadjusted and adjusted models of uterine fibroids and cardiovascular diseases in patients with or without diabetes

Association analysis results between UF and CVDs in diabetes individuals were modeled crude and adjusted for age, BMI, race, education, and family income (Table 3). In diabetes patients, UF patients had a higher odds ratio for CVDs (OR = 2.785, 95% CI = 1.475–5.258, *p* < 0.01). However, in the adjusted model, there is no significance with *p*-values of 0.063. Then, we conducted a logistic regression analysis in patients without diabetes (Table 4). We found that UF patients had a higher odds ratio for CVDs (OR = 2.655, 95% CI = 2.203–3.200, *p* < 0.001). Of note, in the adjudged model, the odds ratio for CVDs in UF patients without diabetes (adjudged for age, BMI, race, education, and income) still has significance (OR = 1.389, 95% CI = 1.124–1.718, *p* < 0.01).

**TABLE 3:** Logistic regression analysis of uterine fibroids and cardiovascular diseases (CVDs) in diabetes individuals: National Health and Nutrition Examination Survey (NHANES) 1999–2006.

	OR	95% CI	*P-value*
**Crude model**			
Uterine fibroids			<0.01
Non-uterine fibroids	Ref.	Ref.	
Uterine fibroids	2.785	1.475–5.258	
**Adjudged model**			
Uterine fibroids			0.063
Non-uterine fibroids	Ref.	Ref.	
Uterine fibroids	1.983	0.964–4.077	
Age, years	1.091	1.050–1.133	<0.001
BMI, kg/m^2^	1.059	1.020–1.099	<0.01
Race			0.128
Mexican American	0.494	0.115–2.121	0.342
Other Hispanic	0.331	0.055–1.990	0.227
Non-Hispanic white	0.981	0.236–4.083	0.979
Non-Hispanic black	1.285	0.310–5.327	0.729
Other race or multi-racial	Ref.	Ref.	
Education			0.303
<High school	Ref.	Ref.	
High school	0.516	0.210–1.267	0.149
>High school	0.596	0.272–1.308	0.197
Annual family income			0.422
<20,000 USD	Ref.	Ref.	
≥20,000 USD	0.771	0.409–1.454	

**TABLE 4:** Logistic regression analysis of uterine fibroids and cardiovascular diseases (CVDs) in patients without diabetes: National Health and Nutrition Examination Survey (NHANES) 1999–2006.

	OR	95% CI	*P-value*
**Crude model**			
Uterine fibroids			<0.001
Non-uterine fibroids	Ref.	Ref.	
Uterine fibroids	2.655	2.203–3.200	
**Adjudged model**			
Uterine fibroids			<0.01
Non-uterine fibroids	Ref.	Ref.	
Uterine fibroids	1.389	1.124–1.718	
Age, years	1.074	1.065–1.083	<0.001
BMI, kg/m^2^	1.067	1.056–1.078	<0.001
Race			<0.001
Mexican American	0.996	0.617–1.607	0.987
Other Hispanic	1.102	0.613–1.981	0.745
Non-Hispanic white	1.333	0.849–2.092	0.212
Non-Hispanic black	1.882	1.182–2.996	<0.01
Other race or multi-racial	Ref.	Ref.	
Education			0.053
<High school	Ref.	Ref.	
High school	1.134	0.892–1.441	0.305
>High school	0.890	0.714–1.110	0.302
Annual family income			<0.05
<20,000 USD	Ref.	Ref.	
≥20,000 USD	0.795	0.659–0.959	

## Discussion

In this cross-sectional study, we investigated the association between CVDs and UF in the general population and diabetes population by using the NHANES 1999–2006 data. We found that UF patients had a higher risk of CVDs, and this association still remained significant after taking into account age, BMI, diabetes, race, education, and family incomes. In addition, we evaluated models limited to women with or without diabetes in the secondary analysis. These models indicated the role of UF in promoting CVDs seems more significant in non-diabetes individuals.

Various evidence suggested similar risk factors or biological mechanisms between fibroids and CVDs, which contain atherosclerosis, hypertension, and hyperlipidemia (13). Previous studies indicated that a high-fat diet is associated with levels of endogenous estradiol, a risk factor for UF (14). However, another study in Japan suggests no significant association between fat intake and UF, while alcohol intake increased the risk of UF (15). The dietary patterns have been proved to be linked with the occurrence of CVDs (16); therefore, it is plausible that they shared similar risk factors in dietary composition. In addition, our previous study suggested a relationship between the dietary inflammatory index and heart failure (17). Another interesting finding is that Simvastatin, a traditional cardiovascular drug, inhibits the Wnt/β-catenin pathway to delay the progress of UF (18). Statin is a kind of drug that is applied in lowing blood lipids, increasing the stability of atherosclerotic plaque and playing a role in anti-inflammation (19). Korkmaz1 et al. reported that uterine leiomyoma was connected with blood lipid profile, insulin resistance, and carotid intima-media thickness (CIMT) in reproductive-aged women in a small sample size study (20). Indeed, hyperlipemia, insulin resistance, and atherosclerosis are essential risk factors for various CVDs. On the contrary, in a cohort with 972 participants, Shannon et al. suggested that the presence of UF was not associated with CIMT and left ventricular mass (21). Nevertheless, our results partly support Korkmaz1’s findings, as the incidence of coronary heart disease, angina, and heart attack are higher in UF patients in our study.

Another issue that should not be ignored is the side effects of the treatment of UF. A Norwegian cross-sectional study indicated a larger cumulative probability of CVDs after hysterectomy (22). Thus, this type of operation might affect the occurrence of CVDs. Androgen is also applied in UF for its role in inhibiting the growth of tumors, however, the roles of androgen in CVDs are still controversial (23). In addition, mifepristone as a glucocorticoid antagonist, used in inhibiting the growth of UF, reduced the high density lipoprotein-cholesterol (HDL-C) and high density lipoprotein (HDL) particle concentration (24). However, mifepristone did not affect corticosterone-induced hypertension (25). Indeed, these confounding factors need further basic and clinical perspective epidemiology investigation to clarify their relationships.

On the other hand, diabetes is one of the recognized cardiovascular risk factors, while various antidiabetics have been proved to be beneficial for improving cardiovascular outcomes (26). As expected, the factor of diabetes occupied the highest odds ratio for CVDs in the adjusted model in our study. Thus, we further performed secondary analysis according to the diagnosis of diabetes. Amazingly, in non-diabetes individuals, UF significantly has an OR other than in diabetes individuals. A previous population study from America demonstrated that diabetes is a protective effect of UF regardless of medication type (3); therefore, it might blunt the effect of UF on CVDs. However, this interesting finding still needs further prospective studies to verify.

There are still some limitations in the present study. First, this is an observational study that obtain diagnosis information through interviews. Second, considering the censoring data on smoking in this study, we do not analyze the influence of smoking in the logistic model. Perhaps due to the societal attribute of female, more than 50% of the female participants refused to answer the question about smoking. Nevertheless, there was no significant difference in smoking status between UF patients and non-UF individuals in this study. In addition, a previous study showed no significant difference in smoking between UF and healthy individuals (2). Third, hypertension patients account for the highest proportion of CVDs in the study, which might contribute to a bias.

## Conclusion

UF might increase the risk of CVDs, while this role seems more harmful in non-diabetes individuals rather than in diabetes. However, further prospective study or animal research is required to confirm their relationship and unveil the underlying mechanism.

## Data availability statement

The datasets presented in this study can be found in online repositories. The names of the repository/repositories and accession number(s) can be found below: https://wwwn.cdc.gov/nchs/nhanes/Default.aspx.

## Ethics statement

The studies involving human participants were reviewed and approved by the Participants provided written informed consent and the process were approved by the National Center for Health Statistic’s Research Ethics Review Board. The present study is based on a secondary date analysis which lacked personal identifiers and does not need institutional reviewing. The patients/participants provided their written informed consent to participate in this study.

## Author contributions

ZL, HL, and BL designed the experiments and wrote the manuscript. BL, ZL, YZ, FL, and ZYu collected and analyzed the data. BL, ZL, ZYu, LH, and ZYa organized the figures and tables. HL, ZL, LH, and ZYa helped with data interpretation. BL and ZL critically reviewed the manuscript. All authors contributed to the article and approved the submitted version.
